# Female Papillary Thyroid Cancer Survivors Are at Increased Risk of Adenomyosis and Endometrial Hyperplasia

**DOI:** 10.7759/cureus.38989

**Published:** 2023-05-14

**Authors:** Tetiana Tatarchuk, Mykola Tronko, Panagiotis Anagnostis, Liudmyla Kalugina, Natalia Pedachenko, Anna Danylova, Tetiana Kuchmenko

**Affiliations:** 1 Obstetrics and Gynecology, Institute of Pediatrics, Obstetrics and Gynecology of the National Academy of Medical Sciences of Ukraine, Kiev, UKR; 2 Diabetes and Endocrinology, State Institution “V.P. Komisarenko Institute of Endocrinology and Metabolism of National Academy of Medical Science, Kiev, UKR; 3 Diabetes and Endocrinology, Academic Orthopaedic Department, Papageorgiou General Hospital, Thessaloniki, GRC; 4 Diabetes and Endocrinology, Center of Orthopaedic and Regenerative Medicine - Center of Interdisciplinary Research and Innovation, Aristotle University Medical School, Thessaloniki, GRC; 5 Obstetrics and Gynaecology, Institute of Pediatrics, Obstetrics and Gynecology of the National Academy of Medical Sciences of Ukraine, Kiev, UKR

**Keywords:** endometrial hyperplasia, adenomyosis, radioactive iodine, papillary thyroid cancer, thyroid cancer survivors

## Abstract

Purpose: Thyroid cancer (TC) is the most common endocrine cancer worldwide, affecting mainly women of reproductive age. However, no data exist about its association with endometrial or uterine disorders. This study aimed to assess the risk of hyperproliferative pathology of the reproductive system in female ТС survivors.

Methods: This was a cross-sectional study of female patients aged 20-45 years diagnosed with papillary TC (PTC) from 1994-2018. Age-matched females with normal thyroid structures served as controls.

Results: One hundred and sixteen patients (mean age 36.7±61 years) and 90 age-matched controls were included. PTC survivors demonstrated an increased risk for adenomyosis [odds ratio (OR) 2.5, 95% confidence interval (CI) 1.3-4.8] and endometrial hyperplasia (OR 3.9, 95% CI 1.1-14.3), compared with controls. The risk for adenomyosis was higher after the ten post-operative years (OR 5.3, 95% CI 2.29- 12.05) than during the first 5-10 years (OR 2.3, 95% CI 1.02-5.10) and increased with the number of RAI courses and the degree of TSH suppression. The risk of endometrial hyperplasia was most evident during the first five years post-thyroidectomy (OR 6.0, 95% CI 1.4-25.5), especially in patients with TSH <0.1 mU/L (OR 6.8, 95% CI 1.4-33.28) No difference in uterine leiomyomas or endometrial polyps was found between PTC survivors and controls.

Conclusions: Female PTC survivors are at increased risk of endometrial hyperplasia and adenomyosis compared with those with normal thyroid structures.

## Introduction

Thyroid cancer (TC) is the most common endocrine cancer, with a rapidly increasing incidence worldwide [1]⁠. It mainly affects women of reproductive age, ranking as the fifth most frequent type of cancer in females worldwide [2]⁠. Papillary TC (PTC) constitutes the “lion’s share”, accounting for approximately 80% of TC cases [3]⁠. Total or subtotal thyroidectomy and, in selected cases, lobectomy is the cornerstone of treatment for PTC [4]. In cases of high risk of relapse, radioactive iodine (RAI) is also administered as adjuvant therapy to reduce this risk [4]. Depending on the patient’s risk category, further management includes long-term thyroid stimulating hormone (TSH)-suppressive therapy⁠. Such a treatment strategy guarantees a favorable prognosis with a 5-year survival rate in >98% of patients [2]. However, RAI is associated with short and long-term dose-dependent adverse effects, such as sialoadenitis, transient loss of taste or smell, xerostomia, alopecia, and increased risk of hematological abnormalities [5]. Quality of life is also impaired in TC survivors, especially in females [6]⁠⁠.

Current evidence also indicates that during the first five years since TC diagnosis, some pathologies of the female reproductive system, such as inflammatory pelvic organ diseases, ovarian cysts, and menstrual disorders, occur almost twice as frequently compared with the general population [7]. Although the pathogenetic mechanisms are not fully elucidated, non-surgical treatment of TC (i.e., RAI) may significantly contribute to the increased incidence of these disorders [8-10]⁠. Further evidence is needed to clarify the exact effect of TC and its management on female reproductive health.

The present study aimed to investigate the risk of hyperproliferative pathology of the reproductive system in female PTC survivors.

## Materials and methods

Study design

The primary cohort was formed based on the retrospective analysis of the electronic medical database of the State Institution “V.P. Komisarenko Institute of Endocrinology and Metabolism”, which includes medical records of the underlying disease (PTC) regarding the type of surgery, histology, tumor-nodes-metastasis (TNM) stage, RAI administration, the degree and duration of TSH suppression, as well as the clinical and laboratory findings throughout the observation period. Further clinical examination of patients was conducted at the State Institution “Institute of Pediatrics, Obstetrics and Gynecology of NAMS of Ukraine”.

The study included women 20-45 years of age who had undergone thyroidectomy for PTC from 1994 to 2018. PTC survivors with a history of another type of cancer were excluded. Αge-matched women without thyroid gland pathology (i.e., with no thyroid nodules on ultrasound) served as controls. All patients and controls gave written informed consent to participate in the study.

The following parameters were included for analysis: The period from PTC diagnosis to study conduction is classified as 1-5 years, 5-10 years, and >10 years; The number of RAI courses and obtained cumulative Ι131 dose is as follows: one course of RAI treatment with a cumulative dose of Ι131 of 3854.0 (±838.5) MBq (including the Ι131 dose obtained for diagnostic purposes), two RAI courses with a cumulative Ι131 dose of 7356.3 (±1839.9) MBq (including the Ι131 dose obtained for diagnostic purposes) and ≥3 RAI courses of RAI treatment with a cumulative Ι131 dose of 23549.5 (±34496.3) MBq (including the Ι131 dose obtained for diagnostic purposes); The average TSH concentrations, classified as follows: <0.1 mU/L, 0.1-0.4 mU/L, and 0.5 ≥ mU/L

Statistical analysis 

Quantitative parameters were presented per group as mean ± standard deviation values, and qualitative parameters were presented as categorical variables (number of observations). All parameters were analyzed by ANOVA, chi-square, and Mann-Whitney tests for the primary and secondary endpoints for a subgroup. All statistical tests were two-tailed, with the significance threshold at 0.05. The STATA software (Version 14, 2018, USA) was used for the analysis.

## Results

The final cohort included 116 women with a mean age of 36.7± 6.1 years. Ninety women without a nodular thyroid disease served as controls. The mean age of PTC diagnosis was 27.7±8.2 years. The number of patients in groups 1-5, 5-10, and 10 years post-PTC diagnosis was 35, 44, and 37. Patients in the third group were younger at the time of thyroidectomy compared with the other two groups. They were diagnosed at an advanced tumor stage, and, as a result, 100% of them received RAI remnant ablation with 2.9±4.3 RAI courses and a cumulative I131 dose of 10,292 ±6177.2MBq. In the groups, 1-5 years and 5-10 years after PTC diagnosis, 74.3% and 93.2% of PTC cases were diagnosed at an early tumor stage, respectively. The mean number of RAI courses and cumulative I131 dose was 0.9±0.5 and 4236.4±2152.4 MBq in the first group and 1.0±0.4 and 4618.5±1457.5 MBq in the second. The descriptive characteristics of PTC survivors are shown in Table 1.

**Table 1:** Descriptive characteristics of PTC survivors. Abbreviations: PTC: papillary thyroid carcinoma; RAI: radioactive iodine; SD: standard deviation; TSH: thyroid stimulating hormone. Notes: a: p-value <0.05 compared with group A b: p-value <0.05 compared with group B c: p-value <0.05 compared with group C

	Group A (1-5 years from PTC diagnosis)		Group B (5-10 years from PTC diagnosis)		Group C (> 10 years from PTC diagnosis)	
	n	%	n	%	n	%
Age of patients						
20-29 years	4	11.4	12	27.3	2	5.4
30-39 years	19	54.3	14	31.8	22	59.5
40-45 years	12	34.3	18	40.9	13	35.1
Mean age±SD (years)	36.6±5.4		36.1±7.5		37.7±4.7	
Age at the time of thyroidectomy						
< 20 years	0	0	5	11.4	14	37.8
20-29 years	8	22.8	15	34.1	18	48.7
30-39 years	24	68.6	23	52.3	5	13.5
40-42 years	3	8.6	1	2.2	0	0
Mean age±SD (years)	33.3±5.2^c^		28.5±7.7^a^		21.6±7.2^a,b^	
PTC stage at diagnosis						
T1	20	57.1 ^c^	26	59.1 ^c^	6	16.2 ^a,b^
T2	4	11.4 ^c^	4	9.1 ^c^	11	29.7 ^a,b^
T3	11	31.5 ^c^	14	31.8 ^c^	4	10.8 ^a, b^
T4	0	0 ^c^	0	0 ^c^	16	43.3 ^a,b^
Treatment						
Surgery only	9	25.7 ^b c^	3	6.8 ^a^	0	0 ^a^
Surgery plus RAI	26	74.3	41	93.2	37	100
Mean cumulative І^131^ dose±SD (MBq)	4236.4±2152.4 ^c^		4618.5±1457.5 ^c^		10292±6177.2 ^a b^	
Mean TSH±SD (mΙU/L)	0.43±0.37 ^b^		0.26±0.24 ^a^		0.3±0.16	
Mean L-thyroxine dose±SD (µg)	128.4±26.7 ^c^		140.0±30.1		153.0±31.4 ^a^	

**Figure 1:**
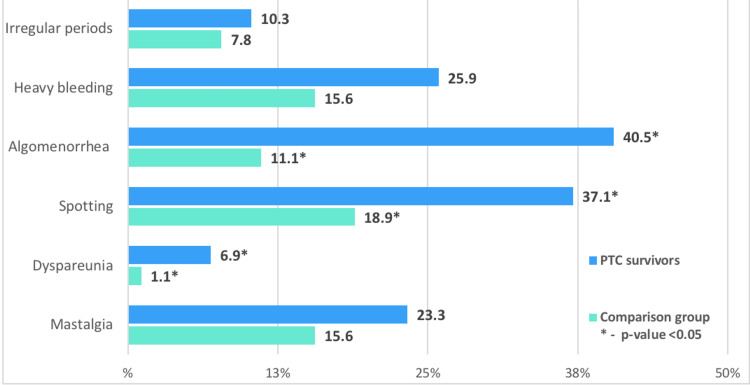
Comparative rates of complaints in PTC survivors and controls.

PTC survivors were more likely to have menstrual disorders, including dysmenorrhea (40.5% vs. 11.1%; р<0.05) and spotting (37.1% vs. 18.9%; р<0.05), compared with controls. Furthermore, 6.9% of PTC survivors complained of dyspareunia, compared with 1.1% of controls (р<0.05). No difference in other symptomatology, such as irregular menstruation and mastalgia, was observed between groups (Figure 1).

**Figure 2:**
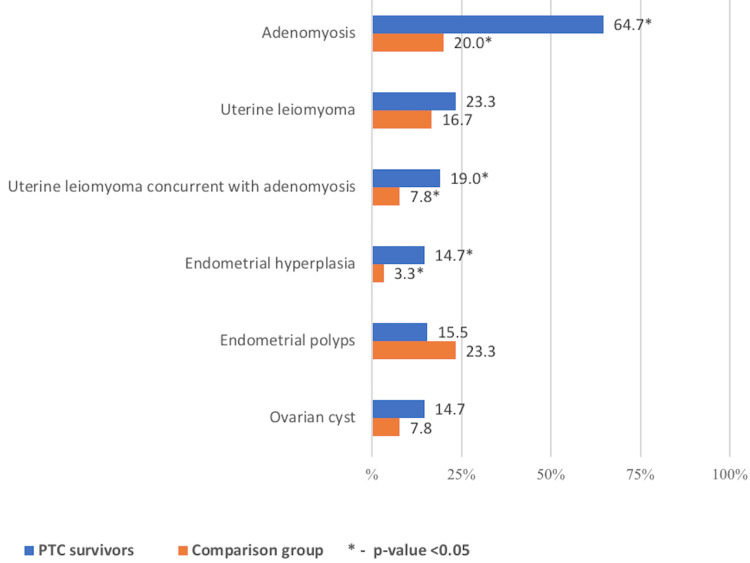
The structure of hyperproliferative pathology of the female reproductive system in PTC survivors compared with controls.

Regarding hyperproliferative pathology of the reproductive system, adenomyosis and uterine leiomyoma concurrent with adenomyosis were more frequently diagnosed in PTC compared with controls [64.7% vs. 20.0% (р<0.05) and 19.0% vs. 7.8% (р<0.05), respectively]. This was also the case for endometrial hyperplasia [14.7% vs. 3.3%, respectively (p<0.05)]. No difference between groups was noticed for other hyperproliferative pathology of the pelvic organs, such as uterine leiomyomas in general, ovarian cysts, and endometrial polyps (Figure 2).

**Figure 3:**
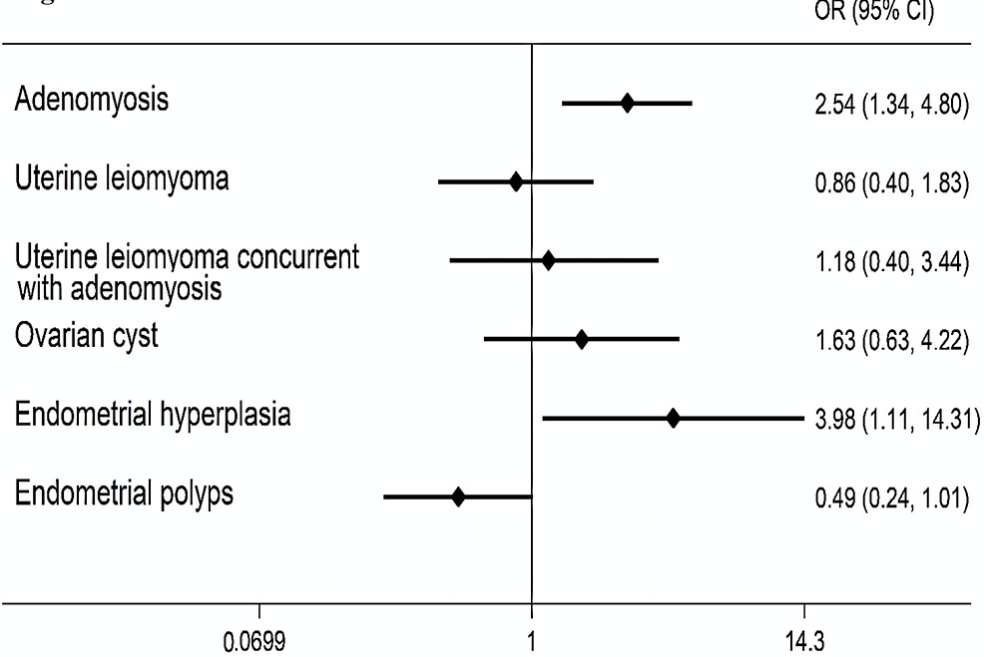
Risk of hyperproliferative pathologies of the female reproductive system among PTC survivors compared with controls.

About the association between PTC and gynecological pathology, PTC survivors demonstrated an increased risk for adenomyosis (OR 2.50, 95% CI 1.34-4.80) and endometrial hyperplasia (OR 3.98, 95% CI 1.11-14.31) compared with controls. There was no increase in the risk of other hyperproliferative pathology (uterine leiomyomas, endometrial polyps, and ovarian cysts) in patients of the primary cohort (Figure 3).

The incidence of adenomyosis showed a gradual increase during the follow-up period after thyroidectomy [OR 2.3 (95% CI 1.02-5.10) for the group of 5-10 years; 5.3 (95% CI 2.29-12.05) for the group of >10 years]. 

The risk for adenomyosis significantly increased with the number of RAI courses [OR 5.33 (95% CI 1.64-17.32 and 8.0 (95% CI 2.17-29.55), for 2 and ≥3 courses, respectively, compared with controls]. It also increased as TSH was suppressed (OR 3.64 (95% CI 1.34-9.89) and 2.51 (95% CI 1.27-4.95) for those with TSH <0.1 and 0.1-0.5 mIU/L, respectively). The risk of endometrial hyperplasia this has also higher in patients with TSH concentrations <0.1 mIU/L (OR 6.82, 95% CI 1.40-33.28) and 1-5 years after PTC diagnosis (OR 6.00, 95% CI 1.41-25.53). No increase was observed with increasing RAI courses. 

Furthermore, there was no effect of the number of RAI courses and the level of TSH suppression on the risk of uterine leiomyoma, endometrial polyps, or ovarian cysts.

## Discussion

This study assessed the risk of hyperproliferative pathology of the female reproductive system in PTC survivors under 45. Female PTC survivors demonstrated a 2.5-fold increased risk for adenomyosis, which increased with the number of RAI courses and TSH suppression therapy. A history of PTC was also associated with a four-fold increased risk for endometrial hyperplasia, which further increased to almost seven-fold in cases with TSH concentrations <0.1 mIU/L.

In general, little data exist on whether the hyperproliferative pathology of the reproductive system, particularly adenomyosis, is a risk factor for TC or vice versa. In particular, an increased risk of TC has been reported in patients with adenomyosis (almost two-fold) [11]⁠ and endometriosis (40% higher, compared with the general population) ⁠[12]. Guenego et al. also reported a two-fold increased risk of TC in women with uterine leiomyomas ⁠[13]. A positive association between TC and uterine fibroids both in pre- [hazard ratio (HR) 1.67, 95% CI 1.22-2.30)] [14] and postmenopausal women (HR 1.72, 95% CI 1.18-2.50) [15] has also been reported by others.

Some pathogenetic mechanisms linking TC with female reproductive pathology can be proposed. First, an elevated ratio of depurinating estrogen-DNA adducts to estrogen metabolites and conjugates, found in women with well-differentiated TC, suggests that initiation of TC arises from the estrogens acting not as hormones but as chemicals that are metabolically activated to the ultimate carcinogenic metabolites, the estrogen-3,4-quinones [16]⁠. In particular, E1 and E2 are oxidized by cytochrome P450 1B1 to the catechol estrogens, 4-OHE1(E2) and then to the estrogen 3,4-quinones [E1(E2)-3,4-Q], which react with DNA to form the 4-OHE1(E2)-1-N3Ade and 4-OHE1(E2)-1-N7Gua adducts [17], which depurinate from DNA, leaving apurinic sites in the DNA that can generate oncogenic mutations [16]⁠. Moreover, animal studies have demonstrated the ability of catechol-estrogen metabolites to exert their biological action by binding to the cytosolic-murine-binding protein that exhibits high affinity and specificity and, thus, elicits their biological responses independently of classical estrogen receptors [18]. As a result, the significantly elevated ratio of depurinating estrogen-DNA adducts to estrogen metabolites and conjugates may promote the active proliferation of cells with damaged DNA and, therefore, the unbalanced estrogen metabolism can be considered as one of the pathogenetic mechanisms of initiation of both thyroid carcinogenesis and hyperproliferative pathology of the reproductive system. Furthermore, dysregulation of some genes, such as CXCL12, GRIM-19, and Pak4, is associated not only with TC cell proliferation, migration, and invasion but also with the induction of the ectopia and promotion of the spread and localized growth of endometrial cells in the development of adenomyosis [19-21]⁠⁠. Moreover, direct irradiation of the female reproductive tract by RAI accumulated in the lower pelvic organs, such as the urinary bladder or the colon, cannot be excluded.

The increased risk of endometrial hyperplasia in PTC survivors can be explained by the significant fluctuations in thyroid homeostasis, namely the transition from euthyroidism to hypothyroidism followed by a long-term state of exogenous thyrotoxicosis, after thyroidectomy for PTC. These changes in thyroid homeostasis, leading to disturbances in the synthesis, transport, and metabolism of sex-steroid hormones, may be linked to an increased risk of hyperproliferative pathology of the reproductive system [22,23].

It was previously reported that TSH and thyroid hormones are involved in the physiology of the endometrium and ovaries through direct interaction with specific receptors, independently of the hypothalamic-pituitary-thyroid system [24-26]⁠. Furthermore, the expression of thyroid hormones and enzymes from the thyroid hormone metabolism in the endometrium of healthy women has been reported [24-26]. More specifically, TSH has a dose-dependent effect by acting as a proliferative and pro-oxidant factor on the endometrium of healthy women and the ectopic and eutopic endometrium of women with endometriosis [26]. Notably, an increase in T3 and T4 concentrations leads to the proliferation of ectopic endometrial cells and an increase in the concentration of reactive oxygen species [26]⁠.

Certain limitations of the present study should be acknowledged, such as its cross-sectional design and the relatively small sample size, which limit the generalization of our findings. Furthermore, this study was limited to PTC. Therefore, the association between other types of TC, such as follicular and medullary carcinomas, and the pathology of the female reproductive system needs to be further investigated.

## Conclusions

In conclusion, female PTC survivors have an increased risk of adenomyosis and endometrial hyperplasia. The former further increases cases with multiple RAI courses and TSH suppression to <0.1 mIU/L. The latter also increases with the degree of TSH suppression. These findings highlight the importance of a detailed examination of this group, affecting pregnancy planning and necessitating the development of specific strategies to prevent the development of neoplastic processes of the female reproductive system. In any case, further research is needed to justify the inclusion of PTC as a risk factor for these pathologies.
